# External disturbances impact helminth–host interactions by affecting dynamics of infection, parasite traits, and host immune responses

**DOI:** 10.1002/ece3.5805

**Published:** 2019-11-06

**Authors:** Isabella M. Cattadori, Ashutosh K. Pathak, Matthew J. Ferrari

**Affiliations:** ^1^ Center for Infectious Disease Dynamics The Pennsylvania State University University Park PA USA; ^2^ Department of Biology The Pennsylvania State University University Park PA USA; ^3^ Department of Infectious Diseases College of Veterinary Medicine The University of Georgia Athens GA USA

**Keywords:** anthelmintic treatment, helminths, immune response, parasite traits, rabbit, single and dual infections

## Abstract

1. External perturbations, such as multispecies infections or anthelmintic treatments, can alter host–parasite interactions with consequences on the dynamics of infection. While the overall profile of infection might appear fundamentally conserved at the host population level, perturbations can disproportionately affect components of parasite demography or host responses, and ultimately impact parasite fitness and long‐term persistence.
2. We took an immuno‐epidemiological approach to this reasoning and examined a rabbit–helminth system where animals were trickle‐dosed with either one or two helminth species, treated halfway through the experiment with an anthelmintic and reinfected one month later following the same initial regime. Parasite traits (body length and fecundity) and host immune responses (cytokines, transcription factors, antibodies) were quantified at fixed time points and compared before and after drug treatment, and between single and dual infections.
3. Findings indicated a resistant host phenotype to *Trichostrongylus retortaeformis* where abundance, body length, and fecundity were regulated by a protective immune response. In contrast, *Graphidium strigosum* accumulated in the host and, while it stimulated a clear immune reaction, many genes were downregulated both following reinfection and in dual infection, suggestive of a low host resistance.
4. External perturbations affected parasite fecundity, including body length and number of eggs in utero, more significantly than abundance; however, there was no consistency in the parasite‐immune relationships.
5. Disentangling the processes affecting parasite life history, and how they relate to host responses, can provide a better understanding of how external disturbances impact disease severity and transmission, and how parasites strategies adjust to secure persistence at the host and the population level.

## Graphical abstract

External perturbations can alter host–parasite interactions with consequences on the dynamics of infection. We took an immuno‐epidemiological approach and examined a rabbit–helminth system where animals were trickle‐dosed with either one or two helminth species, treated halfway through the experiment with an anthelmintic and reinfected one month later following the same regime. Anthelmintic and the infection with the second species affected parasite fecundity more significantly than abundance; however, there was no consistency in the parasite‐immune relationships.

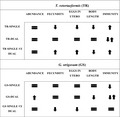

## 1 INTRODUCTION

Variation in host susceptibility to infections plays a fundamental role in the ecology and evolution of infectious diseases. By affecting parasite establishment and onward transmission, individual variation has been shown to affect disease dynamics and outbreaks (Boots, White, Best, & Bowers, 2012; Lloyd‐Smith, Schreiber, Kopp, & Getz, 2005). Some of the drivers that generate this variation are predictable and related to the intrinsic characteristics of the host, mainly genetic, age and sex, or of the parasite such as, the diversity in virulence among strains (Wilson et al., 2002). However, heterogeneities in infection and transmission more often emerge from the complex interactions between the parasite, the host, and external perturbations. The infection with a second parasite species or the exposure to environmental constraints, including the effect of human interventions, like drug treatments or vaccination, is an example of these perturbations.

At the host population level, variations in parasite load among hosts can be captured by a few distinct dynamics and fundamental host responses. A convex relationship between parasite abundance and host age—or time since initial infection—is indicative of resistance, where abundance decreases in older hosts due to a protective immune response (Allen & Maizels, 2011; Duerr, Dietz, & Eichner, 2003; Maizels, Hewitson, & Smith, 2012; Woolhouse, 1992). In contrast, the constant accumulation of parasites as host ages suggests an inadequate control (Hudson & Dobson, 1995), either because individuals have low resistance or tolerate the infection (Best, White, & Boots, 2008; Råberg, Sim, & Read, 2007). External perturbations can change the shape of these relationships and further contribute to heterogeneities in infection. For example, following anthelmintic treatment, a slower accumulation of parasites during reinfections in endemic areas is consistent with a more effective immune response (Jia, Melville, Utzinger, King, & Zhou, 2012). Similarly, infections with a second parasite species can either suppress or enhance the abundance of a first species by altering the immune response and the susceptibility to future infections (Druilhe, Adama, & Sokhna, 2005; Elias, Akuffo, & Britton, 2006; Graham, 2008). Any change caused by external disturbances on the way parasites interact with the host is also expected to impact parasite growth and fecundity. In the instance of reinfection after anthelmintic, a slow accumulation of parasites can be associated with worms that are stunted or have low fecundity (Jia et al., 2012). Likewise, under harsh climatic conditions, parasites can arrest development once in the host and resume it to full maturation and reproduction as conditions improve, a phenomenon well described in small ruminants and a few wildlife species (e.g., red grouse) (Dobson & Hudson, 1992; Gibbs, 1986).

While external disturbances are regularly documented in host–parasite interactions, how they impact parasite dynamics and whether they equally affect parasite abundance and traits (i.e., growth and fecundity) it is not fully understood. Disentangling the conditional reactions of the parasites to these perturbations and how they adjust their life‐history strategy (Jones, 2008; Reznick, Bryant, & Bashey, 2002) is important for explaining how parasites maximize transmission while securing long‐term persistence in the host populations. In the current study, we synthesized fundamental concepts from population dynamics with life‐history principles to assess quantitative changes in parasite variables (i.e., abundance, fecundity, body length, and eggs in utero) and related host immune responses, following perturbations with an anthelmintic and infection with a second parasite species. As a study case, we used laboratory infections of two gastrointestinal helminths, *Trichostrongylus retortaeformis* and *Graphidium strigosum*, in the European rabbit (*Oryctolagus cuniculus*). If the host immune response is the main limitation to parasite accumulation and fecundity, we predict lower abundance and/or fecundity during reinfections after the anthelmintic because of immune memory and faster regulation. However, if there is a lack of or weak immune control, then we would expect to see no changes in parasite dynamics or traits in the reinfection. Then again, if parasites actively avoid/resist the host immune response, we predict changes in abundance and/or traits indicating the cost to the parasite of avoiding host defenses. Finally, given that the two parasites are closely related (Audebert & Durette‐Desset, 2007) and colonize different parts of the gastrointestinal tract, we expect interference mediated by the immune response in rabbits with dual infections, with higher interference if the parasite is under a stronger immune regulation.

## 2 METHODS

### 2.1 The host–parasite system

*Trichostrongylus retortaeformis* colonizes the small intestine and concentrates in the duodenum, while *Graphidium strigosum* inhabits the stomach and primarily the fundus (Murphy, Nalpas, Stear, & Cattadori, 2011). Both helminths have a direct life cycle: Host infection is by ingestion of herbage contaminated with infective larvae and parasite eggs are shed in the environment with hosts' feces. In the laboratory, we showed that *T. retortaeformis* is regulated by an anti‐inflammatory (type 2) immune reaction, while this same type of response does not appear to control *G. strigosum* (Murphy et al., 2011; Murphy, Pathak, & Cattadori, 2013; Takar, Pathak, Murphy, Albert, & Cattadori, 2012). Our field studies also showed that infections with the second helminth can alter the abundance, fecundity (number of eggs in utero/adult female body length), and shedding (number of eggs in feces) of the first parasite (Cattadori, Boag, & Hudson, 2008; Cattadori et al., 2014). Laboratory findings were based on a single‐dose infection, while long‐term field studies included heterogeneities such as hosts of different ages, sexes, and parasite loads. The current laboratory study has a more complex design in that it combines two external disturbances, that is, anthelmintic treatment and infection by a second parasite species, and is based on weekly trickle doses to reflect the natural infections in the field. The current study also quantifies parasite abundance, body length, and eggs in utero over the course of the experiment and investigates a much larger immune profile by including cytokines and transcription factors as well as specific and total antibodies.

### 2.2 Experimental design and sampling

We implemented single and dual laboratory infections where animals were treated with an anthelmintic halfway through the trials (Figure S1). Groups of 58 New Zealand white, 2 months old, male rabbits (*Oryctolagus cuniculus*) were oral gavaged with either 400 *T. retortaeformis* or 100 *G. strigosum* infective third stage larvae (*N* = 36), and control animals (*N* = 22) were sham inoculated with the same volume of water. Animals were trickle‐dosed every 7 days and the first dose marked day 0 of the infection. The different doses were estimated from the burdens of infection observed in the field (Cattadori, Boag, Bjørnstad, Cornell, & Hudson, 2005; Cattadori et al., 2008). The weekly frequency was chosen to mimic host feeding rate while avoiding stressing the animals too frequently. At 60 and 75 days postinitial infection for *T. retortaeformis* and *G. strigosum*, respectively, animals were treated orally for five consecutive days with the broad‐spectrum anthelmintic fenbendazole at dosage adjusted by animal's body mass (5 mg fenbendazole/kg body weight, based on a 10% suspension (100 mg/ml), Panacur, Intervet Inc., USA). Infection and animal sampling were suspended for one month (including the days of treatment) to allow the waning of the immune response and subsequently resumed following the same experimental regime. The different onset of the treatments takes into account the slower development of *G. strigosum* compared to *T. retortaeformis* (prepatent period: ~40 and ~12 days, respectively) and is a reasonable compromise with the technical limitations of running a long‐term experiment with a large number of animals. The anthelmintic successfully removed all the parasites except for a few *G. strigosum* found in four animals after treatment in the single infection (mean ± *SE* = 12.5 ± 9.75). For the dual infection, we followed the same procedures but *T. retortaeformis* initial infection was shifted 15 days later than *G. strigosum* initial infection to mimic a natural lag in the infection onset; both helminths were then administered from the third week of the trial (i.e., 3rd dose for *G. strigosum* and 1st for *T. retortaeformis*). A dual infection, where *G. strigosum* was shifted relative to *T. retortaeformis*, was also planned but not implemented due to time constraints. The sampling of rabbits and the onset of the anthelmintic treatment in dual infected rabbits matched the single infections. This was possible because of we shifted *T. retortaeformis* infection 2 weeks later the start of the experiment and *G. strigosum* treatment started at day 75, which coincides with the treatment of *T. retortaeformis* at day 60 (Figure S1). Groups of six animals (four infected and two controls) were removed at fixed days (days postinfection, DPI) and the remaining animals were weekly dosed until were sacrificed (Figure S1). Sections of duodenum for *T. retortaeformis* and fundus for *G. strigosum* were collected from each animal to quantify parasite and immune variables, following methodologies described extensively elsewhere (Cattadori et al., 2014; Chylinski, Boag, Stear, & Cattadori, 2009; Murphy et al., 2011, 2013). Finally, an initial group of four control animals (in addition to the 58) was removed at day 0 to determine baseline immune metrics from collected tissues. All animal procedures were approved by the Institutional Animal Care and Use Committee of the Pennsylvania State University, and all experiments were performed in accordance with relevant guidelines and regulations.

### 2.3 Immuno‐parasitology

Parasite abundance was estimated based on aliquots (2.5 ml) for *T. retortaeformis* and total counts for *G. strigosum*. A subsample of worms (minimum 50 worms for each sex of each species from every rabbit) was collected at random to quantify body length and number of eggs in female's utero (Chylinski et al., 2009).

Expression of genes encoding cytokines and transcription factors was estimated from tissue of the duodenum or fundus of every animal. The genes selected are involved in regulating various arms of the immune system in mammals, and among these arms, the following were quantified: IL4, IL5, IL13, and GATA3 are associated with a type 2 anti‐inflammatory response; IFNγ and Tbet contribute to the type 1 inflammatory reaction; Foxp3, TGFβ, and IL10 are indicative of a tolerogenic protective response, and RORγT is part of the Th17 inflammatory response. We also quantified the expression of genes involved in mucus production MUC2, MUC5AC as they could contribute to parasite expulsion; HPRT was used as housekeeping gene. Nucleic acid sequences of the primers and probes (final primer and probe concentrations: 450 and 125 μM, respectively) and procedures are reported in our previous work (Cattadori et al., 2016; Murphy et al., 2011). Unless stated otherwise, all reagents and equipment were purchased from Thermo Fisher Scientific and used as directed. The qRT‐PCR technology was performed with the PerfeCTa^®^ qPCR FastMix^®^ II, Low ROX™ kits (Quanta BioSciences Inc.) on an ABI7500 real‐time PCR instrument set in “fast mode” (Pathak, Creppage, Werner, & Cattadori, 2010).

Species‐specific IgA responses were measured with ELISA using mucus collected from the duodenum and fundus and diluted 1:10 and 1:5, respectively (Cattadori et al., 2014). Whole adult worm homogenates were used as antigen (Murphy et al., 2011; Pathak et al., 2010). An in‐house “sandwich” ELISA was also developed to quantify total IgA concentrations in mucus. Optimizations and technique details are reported in our previous study (Cattadori et al., 2016). Antibody data from ELISAs were transformed to optical density (O.D.) index following Murphy et al. (2011) and subsequently used for the analysis.

### 2.4 Statistical analysis

Generalized linear models (GLM, with a negative binomial error distribution) were implemented to examine changes in parasite abundance, as a response, in relation to anthelmintic drug treatment (before vs. after) or type of infection (single vs. dual) as independent categorical variables. Sampling time (DPI, days postinitial infection or reinfection) was included as a pairwise interaction with drug treatment (drug*DPI), and tested as a continuous and categorical variable. This interaction was never significant and was removed with backward deletion in order to select the minimum parsimonious model. The relationship between abundance and immune response was also examined using GLM and selecting the minimum model with the backward approach. Linear mixed effect models (LME) were applied to examine changes in parasite fecundity (eggs in utero/adult female body length), including the single effect of body length or eggs in utero, in relation to drug treatment or infection type. Sampling time was also added as pairwise interaction with drug treatment but was removed because it was not significant. Rabbit ID was included as a random factor (random effect on the intercept). Linear models (LM) were used to examine changes in each immune variable, as a response, in relation to drug treatment or infection type. Count data were log‐transformed if necessary. Gene expression data were corrected to the housekeeping gene and control animals prior to the analyses, following standardized methodologies (Murphy et al., 2011). Additional details on the statistical approach are reported in the results and table legends. Analyses were performed using the software R3.3.1 (https://www.r-project.org/).

## 3 RESULTS

**Figure 1:**
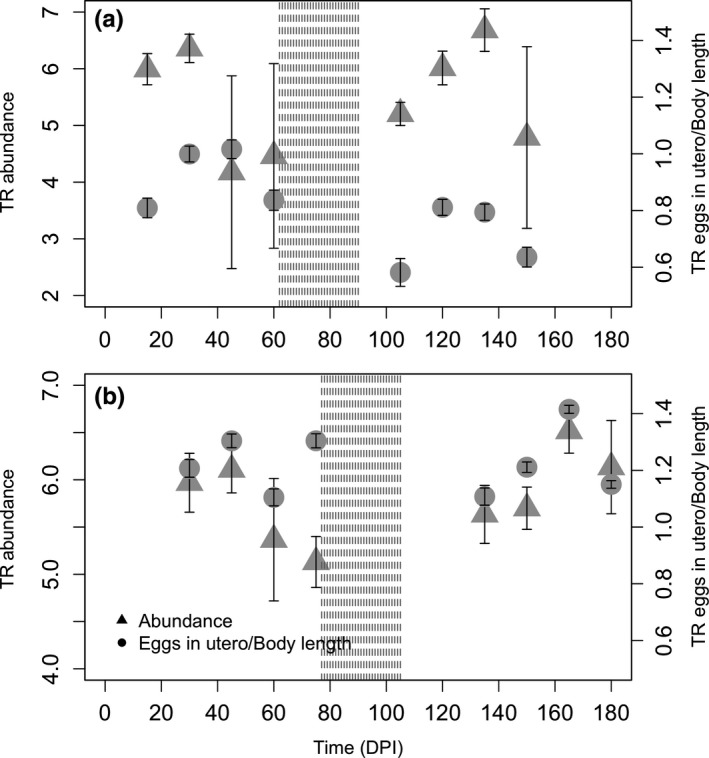
Abundance and fecundity of *T. retortaeformis* (TR) in the duodenum in single (a) and dual infections (b) over time (day postinitial infection). The shaded area represents the period when animals we treated with a 5‐day anthelmintic and left untouched for 30 days. The log(*x* + 1)‐transformed mean and *SE* of the number of parasites/host or parasite fecundity are reported. Triangle = parasite abundance, circle = fecundity

**Figure 2:**
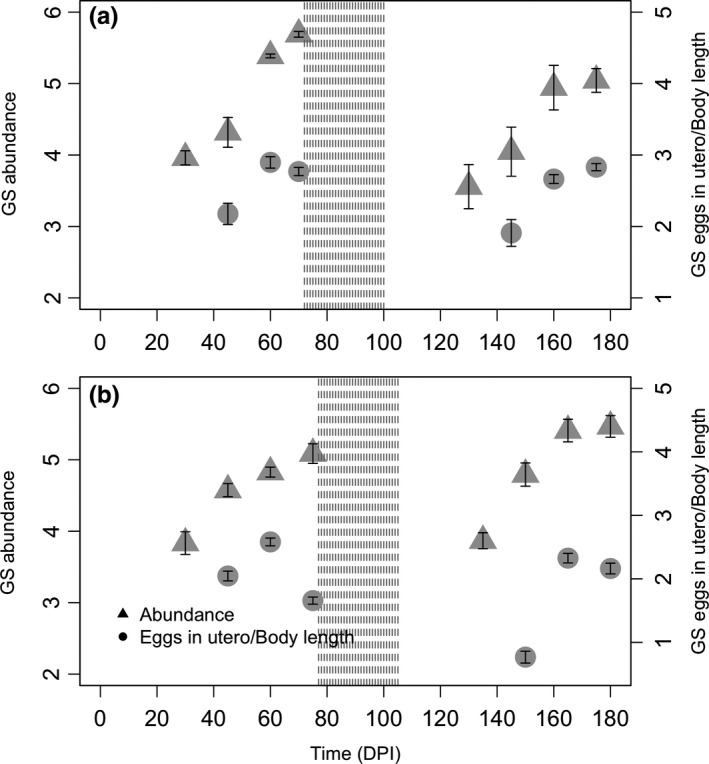
Abundance and fecundity of *G. strigosum* (GS) in the fundus in single (a) and dual infections (b) over time. Additional details are reported in Figure 1

The age‐intensity profiles were consistent with patterns observed in natural populations (Cattadori et al., 2005, 2008, 2014): *T. retortaeformis* but not *G. strigosum* is reduced with time from initial infection (as a proxy of host age) both in the pre‐ and the postdrug treatment (Figures 1 and 2). To clarify how perturbations affected these profiles, we examined parasite abundance and fecundity before and after treatment in single and dual infections, including how these changes were related to the host immune response.

### 3.1 Parasite abundance and traits

In the single infection, *T. retortaeformis* abundance was comparable before and after drug treatment while fecundity was lower in the posttreatment (Table 1, Figure 1a). The lower fecundity was driven by shorter females that carried proportionally fewer eggs (Table 1). A positive, albeit very weak association was found between eggs in utero and abundance (LME coeff. ± *SE* = 0.0006 ± 0.0002, *p* < .01, with random factor rabbit ID (*SD* = 0.445) nested into DPI (*SD* = 0.143)). Based on a visual inspection, the peak of infection was delayed in the posttreatment by one observation period (15 days) while parasite fecundity peaked one observation period earlier (Figure 1a). In the dual infection, *T. retortaeformis* exhibited an analogous pattern (Table 1, Figure 1b): Abundance was comparable before and after drug treatment and, at visual examination, peaked one observation period (15 days) later in the posttreatment. Fecundity (including number of eggs in utero and female body length) did not change after treatment (Table 1). Both eggs in utero (coeff. ± *SE* = 0.0005 ± 0.0001, random factor rabbit ID (*SD* = 0.261) nested into DPI (*SD* = 0.00008)) and female body length (coeff. ± *SE* = 0.0002 ± 0.00005, random factor rabbit ID (*SD* = 0.093) nested into DPI (*SD* = 0.092)) were positively but very weakly associated with abundance (LME for both *p* < .01). When we compared single and dual infections, parasite abundance was similar but fecundity was higher in the dual infection; specifically, females were shorter and carried more eggs in utero (Table 2).

**Table 1:** Summary of linear models between each parasite variable, as a response, and treatment (before and after drug) as independent variable, in single and dual infections

	*T. retortaeformis* Coeff. ± *SE*, *p*		*G. strigosum* Coeff. ± *SE*, *p*	
	Single	Dual	Single	Dual
Abundance	−0.042 ± 0.406	0.272 ± 0.247	−0.405 ± 0.248	0.396 ± 0.196*
Fecundity rabbit ID (*SD*)	−0.428 ± 0.169* 0.427	−0.063 ± 0.207 0.552	−3.420 ± 2.742 6.164	−0.776 ± 2.064 4.737
Body length rabbit ID (*SD*)	−0.604 ± 0.182** 0.466	0.021 ± 0.292 0.812	−0.975 ± 0.772 1.893	−2.68 ± 0.753** 1.792
Eggs in utero rabbit ID (*SD*)	−3.996 ± 1.372** 3.471	−0.842 ± 1.700 4.562	−44.732 ± 44.769 99.770	−32.238 ± 36.351 84.373

Note

We used generalized linear models, with negative binomial error distribution and log‐link, for abundance and linear mixed effect models, with rabbit ID as random factor (SD reported), for fecundity (eggs in utero/body length), body length and eggs in utero. The coeff. ± *SE* and *p*‐value are reported.

* *p* < .05.

** *p* < .01.

*** *p* < .001.

**Table 2:** Summary of linear models comparing parasite variables, as a response, between single and dual infections, as an independent variable

	*T. retortaeformis* Coeff. ± *SE*, *p*	*G. strigosum* Coeff. ± *SE*, *p*
Abundance	−0.658 ± 0.751	0.010 ± 0.165
Fecundity rabbit ID (*SD*)	0.970 ± 0.144*** 0.531	−0.697 ± 0.225** 0.753
Body length rabbit ID (*SD*)	−0.395 ± 0.176* 0.657	−0.087 ± 0.050 0.169
Eggs in utero rabbit ID (*SD*)	6.340 ± 1.200*** 4.400	−0.782 ± 0.242** 0.786

Note

We used generalized linear models, with negative binomial error distribution and log‐link for abundance and linear mixed effect models, with rabbit ID as random factor (*SD* reported), for fecundity (eggs in utero/body length), body length and eggs in utero. The coeff. ± *SE*, *p*‐value and random factor *SD* are reported.

* *p* < .05.

** *p* < .01.

*** *p* < .001.

For *G. strigosum* single infection, both abundance and fecundity were comparable before and after treatment, including numbers of eggs in utero and female body length (Table 1, Figure 2a). In the dual infection, abundance was higher in the posttreatment, whereas fecundity remained unchanged but showed a tendency to decrease later in the infection, both in the pre‐ and posttreatment (Table 1; Figure 2b). In more detail, female body length was shorter in the posttreatment but no changes were found the number of eggs in utero (Table 1), suggesting that shorter females tend to carry proportionately more eggs. A negative relationship was also found between female body length and abundance (LME coeff. ± *SE* = −0.001 ± 0.0004, *p* < .01, with random factor rabbit ID (*SD* = 0.109) nested into DPI (*SD* = 0.298)). Parasite abundance and body length were comparable in single and dual infections but fecundity and number of eggs in utero were lower in the dual infection (Table 2). A summary of these general trends is reported in Figure S2.

### 3.2 Host immune response

**Figure 3:**
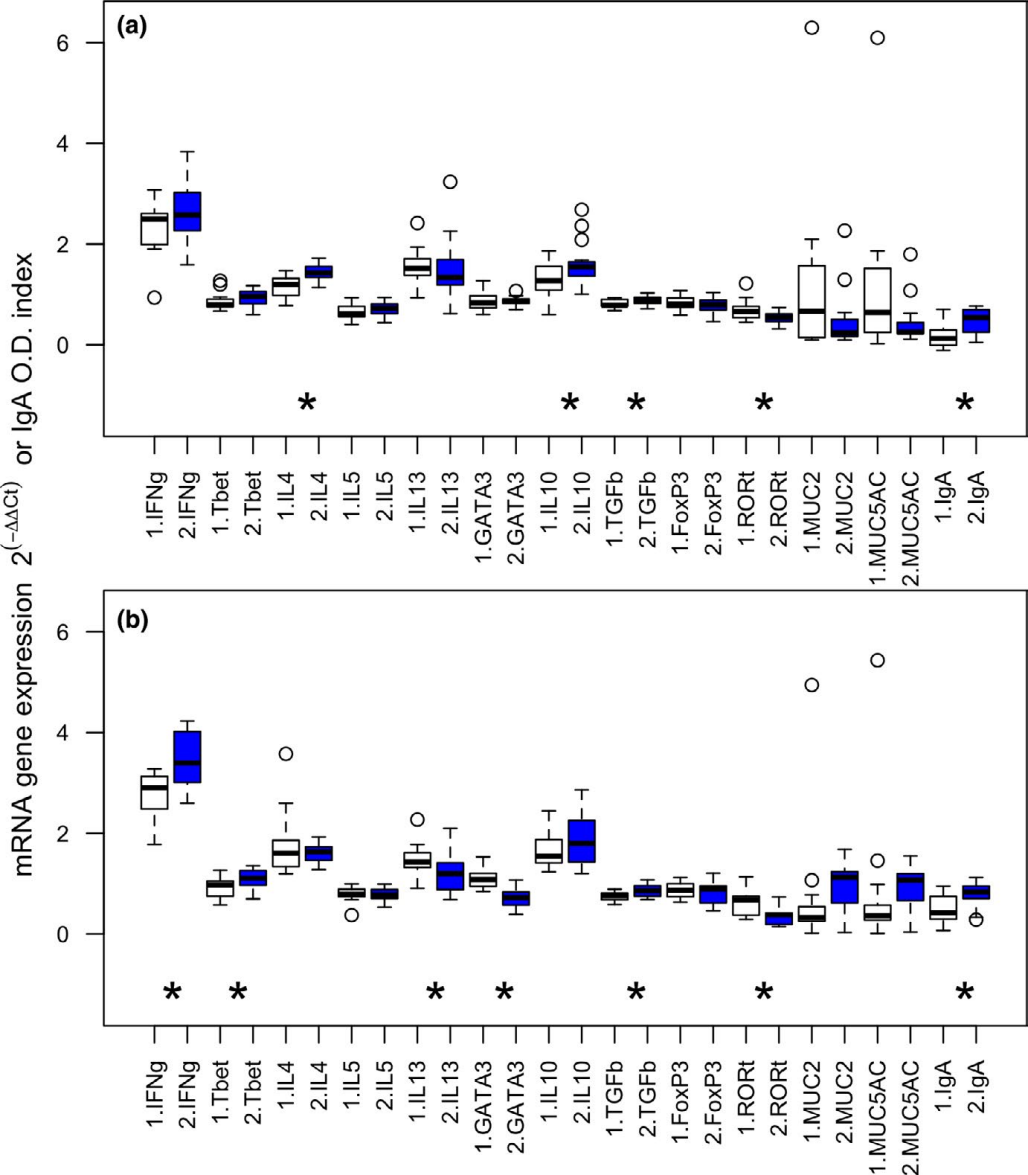
Cytokine and transcription factor gene expression, and antibody optical density (O.D.) index in the duodenum for *T. retortaeformis* before (white) and after (blue) drug treatment in single (a) and dual (b) infections. Variables have been grouped by immune type: type 1 (IFNγ, Tbet), type 2 (IL4, IL5, IL13, GATA3), regulatory T cells (IL10, TGFβ, Foxp3), IL‐17 (RORγT), mucus production (MUC2, MUC5AC), and IgA. Gene expression data were standardized to the housekeeping gene and the control animals; raw Ig data were transformed to optical density index. The median, the 25% and 75% quintiles, the maximum and minimum and outliers of log(*x* + 1)‐transformed data are reported; the star symbol indicates variables with significant differences between pre‐ and posttreatment. Numerical details in Table S1

To identify the immune profiles associated with the patterns of infection, and how they change under perturbations, a number of variables representative of different arms of the immune response were quantified at the site of the infection. In single infections with *T. retortaeformis*, there was a general upregulation in the post‐ relative to the pretreatment phase, notably for IL4, IL10 and TGFβ. On the contrary, Th17 inflammatory RORγT was downregulated in the posttreatment. In the dual infection, the anti‐inflammatory GATA3 and the inflammatory RORγT were downregulated in the posttreatment, while IFNγ, Tbet, and TGFβ were upregulated (Figure 3, Table S1). Although changes in the immune profile (Table S1) were not always significant, it is important to note the consistency of the variables within the same functional immune group, that is, inflammatory, anti‐inflammatory, and regulatory. Among the significant variables, 80% from the single infection and 57% from the dual infection increased in expression after treatment (Table S1). A general upregulation of the immune response was found when comparing dual with single infection, specifically, of the significant variables 71% were upregulated such as IL4, IL5, IFNγ, and IL10 (Figure 3, Table S2).

**Figure 4:**
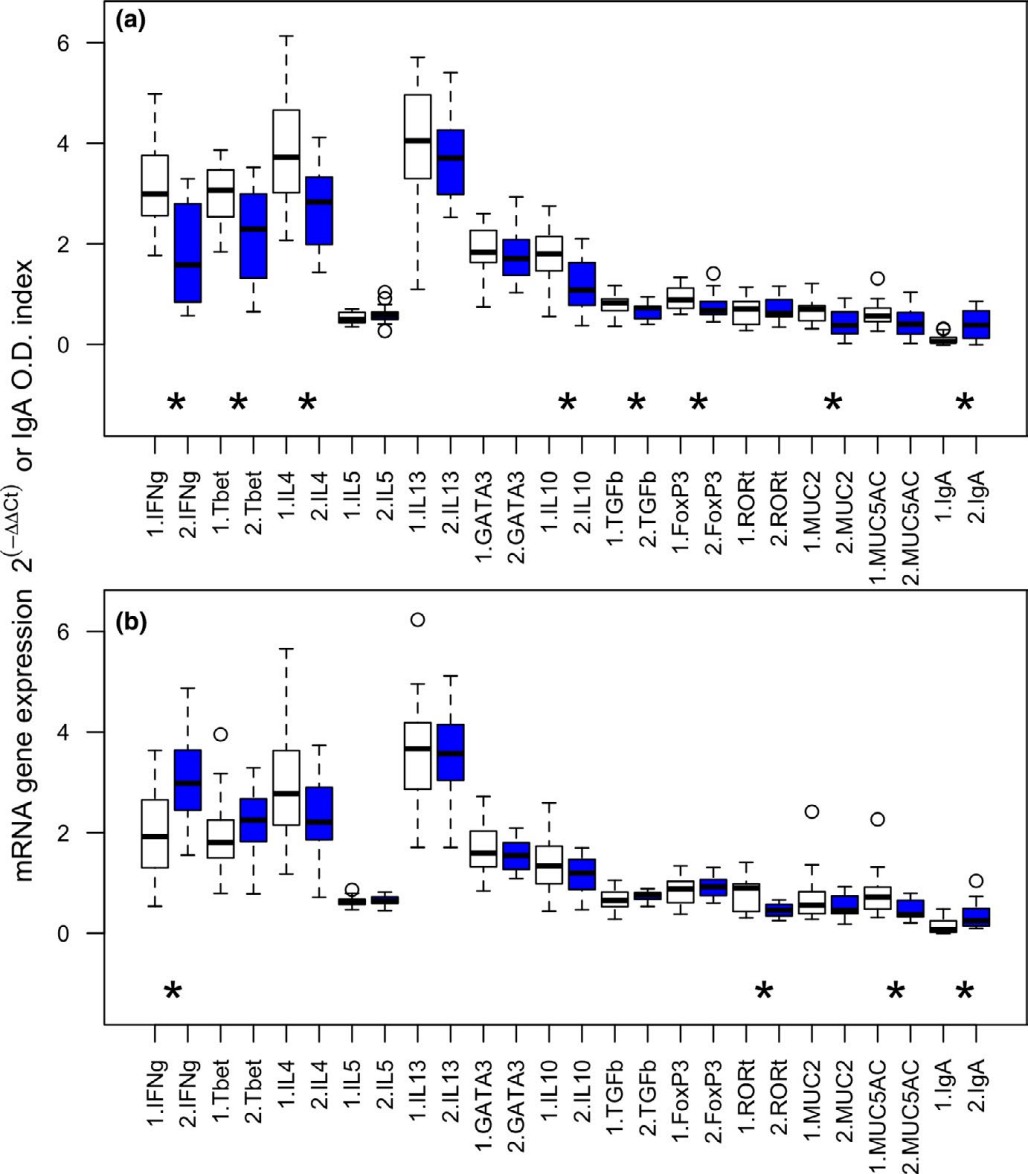
Cytokine and transcription factor gene expression, and antibody optical density index in the fundus for *G. strigosum* before (white) and after (blue) drug treatment in single (a) and dual (b) infections. Additional details are reported in Figure 3

For *G. strigosum*, we found that 77% and 54% of the immune variables from single and dual infections, respectively, were downregulated in the post‐ compared to the pretreatment, although this was not always significant especially in the dual infection (Figure 4). The downregulation affected anti‐inflammatory variables, like IL4, regulatory variables, such as IL10, and inflammatory variables, for example, IFNγ. Among the significant variables, 87% from the single infection and 50% from the dual infection decreased in expression (Table S1). Similarly, a downregulation of the immune response, albeit not always significant, was found in the dual compared to the single infection; significant variables to note were IL4 and Tbet (Table S2).

We also quantified the specific IgA concentration (i.e., species‐specific IgA/total IgA) and for both helminths values were consistently and significantly higher in the posttreatment of single (LM, IgA concentration by treatment for: *T. retortaeformis* coeff. ± *SE* = 0.41 ± 0.163, *p* < .05 and *G. strigosum* coeff. ± *SE* = 0.32 ± 0.094, *p* < .01) and dual infections (*T. retortaeformis:* coeff. ± *SE* = 0.55 ± 0.210, *p* < .05; *G. strigosum:* coeff. ± *SE* = 0.23 ± 0.081, *p* < .01), supporting the stronger antibody reactivity during reinfections. IgA concentration almost doubled in the dual compared to the single infection for *T. retortaeformis* but not for *G. strigosum;* specifically, single and dual infections mean ± SD: for *T. retortaeformis* = 0.476 ± 0.493 and 0.827 ± 0.627; for *G. strigosum* = 0.256 ± 0.302 and 0.246 ± 0.250. A summary of the dominant immure trends against the two helminths is reported in Figure S2.

### 3.3 Relationship between parasites and immune responses

Changes in *T. retortaeformis* abundance and traits were significantly associated with the three arms of the immune response (anti‐inflammatory type 2, inflammatory type 1 or regulatory T cells); however, within the same arm, we found both positive and negative relationships (Table 3). In addition to this, parasite‐immune relationships that were significant in the single infection were not necessarily significant in the dual infection, and vice versa (Table 3). Precisely, abundance was associated both positively (IL10, single infection) and negatively (TGFβ, single infection) with regulatory T cells, positively (GATA3, single infection) and negatively (IL13, dual infection) with type 2 anti‐inflammatory responses, and negatively (IFNγ, dual infection) or positively (Tbet, dual infection) with the inflammatory reaction. The number of eggs in utero and female body length was positively associated with the inflammatory (RORγ and IFNγ, single infection) and T‐cell regulatory (IL10, dual infection) responses and negatively with type 2 (IL13, single infection), T‐cell (TGFβ, dual infection) and type 1 (Tbet, single infection) reactions.

**Table 3:** Summary of linear models between *T. retortaeformis* abundance, number of eggs in utero or female body length, as a response, and immune components as independent variables, for single and dual infections

	Single		Dual
Parasite abundance Coeff. ± *SE*, *p*			
*GATA3*	−1.328 ± 0.679#	*IFNγ*	0.541 ± 0.198**
*IL10*	−0.727 ± 0.255**	*Tbet*	−0.788 ± 0.332*
*TGFβ*	2.433 ± 1.139*	*IL13*	0.318 ± 0.150*
		*IL10*	−0.290 ± 0.152#
		*TGFβ*	1.011 ± 0.361**
		*IgA specific*	−0.149 ± 0.063*
Female body length Coeff. ± *SE*, *p*			
*IFNγ*	−0.036 ± 0.014*	*RORγT*	0.044 ± 0.017*
*Tbet*	0.158 ± 0.029***	*IgA specific*	−0.026 ± 0.008**
*IL13*	0.035 ± 0.011**		
*RORγT*	−0.091 ± 0.023***		
Eggs in utero Coeff. ± *SE*, *p*			
*IFNγ*	−0.028 ± 0.116*	*IL5*	−0.254 ± 0.110*
*Tbet*	0.660 ± 0.246*	*IL10*	−0.116 ± 0.055#
*RORγT*	−0.675 ± 0.191**	*TGFβ*	0.311 ± 0.127*
		*RORγT*	−0.120 ± 0.056*

Note

Gene expression data have been standardized to the housekeeping gene; therefore, negative relationships should be read as positive and positive relationships as negative. We used generalized linear models, with negative binomial error distribution and log‐link, for abundance, while we used linear mixed effect models for eggs in utero and body length, with rabbit ID nested into DPI as random factors. Backward deletion of nonsignificant immune variables was implemented to select, and present, the minimum parsimonious models.

* *p* < .05.

** *p* < .01.

*** *p* < .001.

# .05 < *p*< .055.

Variability between single and dual infections was also found in the relationships of *G. strigosum* with the immune response (Table 4). Abundance was negatively associated with type 2 (IL4, IL5, single and dual infections) and regulatory T cells (FoxP3, single infection), and positively related to the inflammatory response (Tbet, RORγT, dual and single infections). Both the number of eggs in utero and female body length were negatively related to the type 2 reaction (IL4, IL13, GATA3, dual and single infections) and either positively (Tbet, TGFβ, single and dual infections) or negatively (IFNγ, FoxP3, single and dual infections) associated with the inflammatory and T‐cell regulatory responses.

**Table 4:** Summary of linear models between *G. strigosum* abundance, number of eggs in utero or female body length, as a response, and immune components as independent variables, for single and dual infections

	Single		Dual
Parasite abundance Coeff. ± *SE*, *p*			
*IL10*	−0.575 ± 0.097***	*Tbet*	−0.336 ± 0.078***
*FoxP3*	0.617 ± 0.185***	*IL4*	0.249 ± 0.056***
*RORγT*	−0.514 ± 0.135***	*IL5*	1.007 ± 0.276***
Female body length Coeff. ± *SE*, *p*			
*Tbet*	−0.086 ± 0.028**	*IFNγ*	0.073 ± 0.014***
*IL4*	0.043 ± 0.020*	*Tbet*	−0.081 ± 0.027**
*RORγT*	0.079 ± 0.030*	*IL13*	0.068 ± 0.022**
*TGFβ*	0.123 ± 0.059^#^	*IL10*	−0.090 ± 0.032**
*IgA specific*	−0.173 ± 0.054**	*TGFβ*	0.104 ± 0.052^#^
Eggs in utero Coeff. ± *SE*, *p*			
*IL13*	0.272 ± 0.081**	*IFNγ*	0.642 ± 0.126***
*FoxP3*	0.430 ± 0.202*	*Tbet*	−0.887 ± 0.204***
*TGFβ*	−1.207 ± 0.264***	*IL13*	0.300 ± 0.110*
		*GATA3*	0.826 ± 0.256**
		*FoxP3*	0.685 ± 0.285*
		*TGFβ*	−2.042 ± 0.605**

Note

Additional details are reported in Table 2.

## 4 DISCUSSION

We characterized changes in population dynamics and life‐history traits of two gastrointestinal helminths when challenged by an anthelmintic treatment and the presence of the second parasite species. *T. retortaeformis* illustrates the classic sign of limitation in abundance and growth during infection and reinfection of rabbits with one or two parasite species, consistent with host immune mediated control. In contrast, *G. strigosum* shows no clear response to the host immune reaction: Abundance accumulates with the constant exposure of rabbits to infective stages and fecundity is similar before and after anthelmintic treatment in rabbits with single or dual infections.

Rabbits developed a typical type 2 immune reaction; however, the direction and strength was not always consistent between the two parasites or types of infection, a trend also observed for the inflammatory and regulatory responses. Surprisingly, many of the immune genes against *G. strigosum* were downregulated in the postdrug treatment, such as IL4, IL13, GATA 3, and IL 10, supporting the hypothesis that the accumulation of this parasite is associated with a low protective response. While this trend was not always statistically significant, the common behavior among the genes investigated is consistent with the biological relevance of this pattern. Parasitic helminths produce compounds that can manipulate/suppress different components of the host immune response (McSorley & Maizels, 2012; and reviewed in Johnston, McSorley, Anderton, Wigmore, & Maizels, 2014). There is also growing evidence that they can express genes that mimic, as well as stimulate, host tolerogenic functions (Gause, Wynn, & Allen, 2013; Girgis, Gundra, & Loke, 2013; Grainger et al., 2010; Hewitson, Grainger, & Maizels, 2009). We cannot exclude that *G. strigosum* applies some of these strategies to enhance its survival and/or fitness but we do not have clear evidence to support this conclusion. The manipulation of the immune response might not be costly if resources are unlimited; however, rabbits were restricted to 125 g/day of food and needed energy to develop from kittens to adults during the 4‐ to 5‐month experiments. This might have constrained the access of *G. strigosum* to resources and imposed a cost, such as increased mortality or reduced fecundity, if actively manipulating the immune response. We found no evidence of significant parasite mortality or significant changes in fecundity before and after treatment in both types of infection. However, we did find a significant, albeit weak, negative relationship between body length and abundance in dual infected animals, suggesting density‐dependent regulation in parasite growth, and possibly some resource limitation for *G. strigosum*. The further reduction of the type 2 response from single to dual infection, notably for IL4 (IL13 and GATA3 also decrease but not significantly), suggests that this downregulation could become more important in multiple infections with closely related species, for example, by avoiding an excessive cross‐reaction. A recent study showed that wild mice chronically exposed to a large number of pathogens have significantly higher IgE and IgG levels but reduced cytokine reactions than laboratory mice, and explained this trend as a way hosts limit inflammation and maintain immune homeostasis (Abolins et al., 2017). We suggest that the interactions of *G. strigosum* in the stomach could be based on a similar rationale and propose that the host rather than the parasite limits the immune response, probably to reduce a disproportionate reaction and excessive pathology.

The immune profile to *T. retortaeformis* was more variable. The type 2 reaction was upregulated in the post‐ compared to the pretreatment, and this was more apparent in animals with single than dual infections. The type 1 IFNγ and Tbet were generally upregulated. This variability was also apparent in the relationships between immune response and parasite variables. No immune component could consistently explain the parasite patterns by treatment or type of infection. Yet, we suggest that the stunting of *T. retortaeformis* growth from pre‐ to postdrug treatment in single infected hosts could be caused by the significant increase in IL4 and IgA (more below). A similar conclusion, namely, the role of the host immune response on parasite growth and fecundity, was also proposed using a within‐host state‐space model on *T. retortaeformis* single infection data (Ghosh, Ferrari, Pathak, & Cattadori, 2018). The significant increase in IL4 and IL5 from single to dual infection could explain the significant decrease in parasite body length in the later group of hosts. However, the same increase in IL5 could have also positively affected the significant increase in fecundity, namely eggs in utero, in these shorter females. Previous work on filariasis showed that parasite growth and fecundity were enhanced by an IL5‐driven eosinophilia (Babayan, Read, Lawrence, Bain, & Allen, 2010). We can speculate that a similar process could impact parasite reproduction in our system. This pattern is consistent with the classical life‐history trade‐off that predicts higher provision to reproduction in settings with stronger adult mortality (Reznick & Endler, 1982; Stearns, 1992). The stronger type 2 (IL4 and IL5) response and reduced abundance in the dual infection can be considered analogous to increased population mortality due to predation, which in turn could have led to the higher reproductive allocation and more eggs found in utero.

The positive relationship between the type 1 response and the abundance of both parasites is a pattern we also highlighted in our previous work (Murphy et al., 2011). In addition to causing tissue damage, the movement of larvae across the gut wall, as a part of their natural life cycle, could have contributed to bacterial infiltration and the stimulation of an inflammatory reaction, which did not appear to have facilitated the establishment of *T. retortaeformis*. This larval behavior has been described elsewhere (Audebert, Vuong, & Durette‐Desset, 2003) and also confirmed in our recent study where larvae of both helminths were commonly extracted from the gastrointestinal wall of wild rabbits, irrespective of their age, sex, or month of sampling (Van Kuren, Boag, Hrubar, & Cattadori, 2013).

IgA antibodies have been found to be important modulators of the life‐history traits of helminth species (Bleay, Wilkes, Paterson, & Viney, 2007; McCoy et al., 2008; Moreau & Chauvin, 2010; Roach, Else, Wakelin, Mclaren, & Grencis, 1991). For instance, sheep IgA was found to regulate *Teladorsagia circumcincta* growth and eggs in utero (Henderson & Stear, 2006; Stear et al., 2004). IgA was also suggested to be a good indicator of heritable host resistance to helminths (Prada Jiménez de Cisneros et al., 2014; Strain et al., 2002). We previously showed that IgA is important for the control of *T. retortaeformis* intensity of infection (Takar et al., 2012). In the current study, we found that the concentration of specific IgA significantly increased in the posttreatment for both helminths, particularly for *T. retortaeformis*, confirming the stronger immune reaction to, and regulation of, *T. retortaeformis* in contrast to *G. strigosum*.

Variation among individuals probably affected the ability to consistently detect significant relationships between parasites and immune variables. However, by exploring a large immune profile, we were able to identify general trends and key components involved in parasite regulation. We were not able to implement the dual infection, where *G. strigosum* was similarly shifted relative to *T. retortaeformis*, due to time constraints related to the slower development of *G. strigosum*. However, based on the current findings, we would not expect substantial differences for *G. strigosum*, while for *T. retortaeformis*, we would probably see some negative effects on fecundity and growth through a type 2 immune response already stimulated against the first parasite.

Collectively, our system showed that disturbances caused by reinfections after anthelmintic treatment and heterologous infections primarily affected parasite traits rather than abundance, and this was more apparent under stronger immune constraints. By affecting body growth and fecundity, these disturbances can have a strong impact on parasite transmission and disease risk. For example, multispecies infections have been suggested to facilitate the emergence of supershedding individuals. What is generating these cases has proved to be challenging, particularly for parasitic infections where abundance might not be linearly related to fecundity and/or shedding, and variables might not be under the same mechanisms of regulation. More attention should be directed to the understanding of how parasite traits interact with the host immune response and how this impacts onward transmission. This can provide invaluable information on host–parasite coevolution at the individual and the host population level.

## CONFLICT OF INTEREST

The authors have no competing interests.

## AUTHORS' CONTRIBUTION

IMC conceived and designed the study, undertook the statistical analyses, and wrote the manuscript. AKP carried out the animal and molecular work and contributed to manuscript writing. MJF contributed to the work rationale, analyses, and manuscript writing. All authors gave final approval for publication.

## Data Availability

Data are available from the PSU ScholarSphere repository service (https://doi.org/10.26207/50s0-vk90).
