# Supplementary material for: External disturbances impact helminth–host interactions by affecting dynamics of infection, parasite traits, and host immune responses
Source: Ecol Evol. 2019 Nov 6;9(23):13495–505. doi: 10.1002/ece3.5805 (PMC6912924; doi:10.1002/ece3.5805)
Supplement: Supplementary file 1 [file ECE3-9-13495-s001.docx]

Supplementary Material

**External Disturbances Impact Helminth-Host Interactions by affecting Dynamics Of Infection, Parasite Traits And Host Immune Responses**

Isabella M. Cattadori^1,2^, Ashutosh K. Pathak^1,2,3^, Matthew J. Ferrari^1,2^

^1^Center for Infectious Disease Dynamics and ^2^Department of Biology, The Pennsylvania State University, University Park, PA 16082, USA

^3^Department of Infectious Diseases, College of Veterinary Medicine, The University of Georgia, Athens, GA 30602, USA

**Figure S1.** Experimental design of single and dual infections.

**Figure S2.** Summary of the general and dominant trends for *T. retortaeformis* and *G. strigosum*, and related host immune responses.

**Table S1.** Changes in the immune variables before and after drug treatment in single and dual infections.

**Table S2.** Changes in the immune variables between single and dual infections.

**Figure S1**. Experimental design for single and dual infections. Each experiment started at day 0 with the infection of 36 rabbits. Animals received a dose (syringe), of third stage infective larvae (400 *T. retortaeformis*, TR, or 100 *G. strigosum*, GS) every week (Dosing = day of infection). Four infected animals were sacrificed a fixed points (Days Post Infection, DPI = sampling day, except day 0 the starting of the experiment) and the remaining animals (black numbers between rabbits) were weekly dosed until they were sacrificed. At day 60 for TR or 75 for GS all the animals we treated with an anthelmintic for 5 consecutive days and then left alone for a month, including the treatment days (red dotted line). After a month the infection restarted following the dosing and sampling design of the pre-treatment. For the co-infection the first two doses (first two weeks) were GS only, from the third week (3rd dose) both helminth species were administered. In the dual infection the sampling of rabbits and treatment match with the single infection of each parasite because we shifted TR infection 2 weeks later the start of the experiment and GS treatment starts at day 75. First row: *T. retortaeformis* single infection (TR), second row: *G. strigosum* single infection (GS) and third row: dual infection (TR+GS).

**Figure S2.** Overall general trends for *T. retortaeformis* and *G. strigosum*, and related host immune responses, when compared by: i- before and after anthelmintic treatment for single and dual infection (first 2 rows) and ii- between the two types of infections (single *vs* dual) (third row). For the immune response one arrow depicts the common trend while two arrows indicate the lack of a dominant pattern where some variables have a positive other a negative trend. Symbols: equal = no variation, arrow pointing up = increase, arrow pointing down = decrease.

**Table S1.** Summary of linear models between each immune variable (log-transformed), as a response, and drug treatment (before *vs* after) as independent variable, in single and dual infections. Gene expression increases from pre- to post-treatment for positive values, while decreases for negative values. Gene expression data have been standardized to the housekeeping gene and the controls. *=p<0.05, **=p<0.01, ***=p<0.001, #=0.05<p<0.055.

|  | *T. retortaeformis*  Coeff.±S.E., p | | *G. strigosum*  Coeff.±S.E., p | |
| --- | --- | --- | --- | --- |
|  | ***Single*** | ***Co-infection*** | ***Single*** | ***Co-infection*** |
| *IFNγ* | 0.322±0.203 | 0.690±0.175*** | -1.388±0.337*** | 1.136±0.321** |
| *Tbet* | 0.082±0.060 | 0.166±0.068* | -0.751±0.282* | 0.268±0.271 |
| *IL4* | 0.263±0.065*** | -0.147±0.159 | -1.078±0.352** | -0.593±0.350 |
| *IL5* | 0.061±0.048 | -0.014±0.050 | 0.082±0.055 | 0.020±0.037 |
| *IL13* | -0.056±0.175 | -0.245±0.122# | -0.207±0.387 | -0.093±0.358 |
| *GATA3* | -0.014±0.052 | -0.385±0.073*** | -0.112±0.171 | -0.113±0.145 |
| *IL10* | 0.319±0.138* | 0.2467±0.156 | -0.600±0.195** | -0.168±0.164 |
| *FoxP3* | -0.042±0.051 | -0.052±0.066 | -0.173±0.086# | 0682±0.081 |
| *TGFβ* | 0.066±0.029* | 0.119±0.051* | -0.136±0.067# | 0.064±0.060 |
| *RORγT* | -0.153±0.060* | -0.253±0.080** | 0.052±0.090 | -0.316±0.093** |
| *MUC2* | -0.710±0.410 | 0.267±0.3171 | -0.263±0.098* | -0.199±0.147 |
| *MUC5AC* | -0.713±0.382 | 0.164±0.345 | -0.171±0.101 | -0.343±0.129* |
| *IgA specific* | 0.300±0.087** | 0.269±0.094** | 0.295±0.078*** | 0.212±0.076* |

**Table S2.** Summary of linear mixed effect models between every immune variable (log-transformed), as a response, and type of infection (single *vs* dual) as independent variable, for both helminths. Anthelmintic treatment is included as a random factor (S.D. is reported) to take into account the initial conditions of the treatment (i.e. intercept crossing the y axis). Gene expression increases from single to dual infection for positive values, while decreases for negative values. Gene expression data have been standardized to the housekeeping gene and the controls. *=p<0.05, **=p<0.01, ***=p<0.001, #=0.05<p<0.055.

|  | *T. retortaeformis*  Coeff.±S.E., p, S.D. | *G. strigosum*  Coeff.±S.E., p, S.D. |
| --- | --- | --- |
| *IFNγ* | 0.618±0.136***, 0.234 | 0.087±0.280, 2e-5 |
| *Tbet* | 0.119±0.045*, 0.053 | -0.502±0.205*, 3e-5 |
| *IL4* | 0.371±0.088***, 5e-6 | -0.619±0.250*, 0.380 |
| *IL5* | 0.096±0.036**7e-7 | 0.0816±0.033*, 0.010 |
| *IL13* | -0.167±0.107, 0.010 | -0.214±0.260, 2e-5 |
| *GATA3* | 0.0401±0.051, 0.093 | -0.214±0.111, 9e-6 |
| *IL10* | 0.312±0.104***, 0.121 | -0.239±0.130, 0.169 |
| *FoxP3* | 0.039±0.041, 2e-6 | 0.038±0.060, 4e-6 |
| *TGFβ* | -0.033±0.027, 0.039 | -0.023±0.047, 2e-6 |
| *RORγT* | -0.121±0.050*, 0.095 | -0.075±0.069, 0.045 |
| *MUC2* | -0.018±0.264, 2e-5 | 0.070±0.089, 0.099 |
| *MUC5AC* | 0.059±0.261, 3e-5 | 0.103±0.083, 0.115 |
| *IgA specific* | 0.314±0.064***, 0.135 | -0.006±0.055, 0.121 |
